# Evaluating Haversian canal-to-osteon ratios for reconstructing bone remodelling activity: balancing precision and efficiency in data collection

**DOI:** 10.1007/s00774-026-01747-7

**Published:** 2026-08-10

**Authors:** Karen M. Cooke, Isabella Pearce, R. Claire Aland, Julien Louys, Patrick Mahoney, Justyna J. Miszkiewicz

**Affiliations:** 1 School of Social Science, University of Queensland, St Lucia, QLD Australia; 2 School of Biomedical Sciences, University of Queensland, St Lucia, QLD Australia; 3 Australian Research Centre for Human Evolution, Griffith University, Nathan, QLD Australia; 4 School of Natural Sciences, University of Kent, Canterbury, UK; 5 Earth, Life, Time Group, Naturalis Biodiversity Center, Leiden, The Netherlands

**Keywords:** Secondary osteon, Haversian canal, Haversian systems, Histomorphometry

## Abstract

**Introduction:**

In cortical bone histology, the ratio of Haversian canal and secondary osteon areas reflects bone resorption and formation during metabolic events executed by Basic Multicellular Units (BMUs). Area data can be pooled where they are collected independently, and the ratio calculated from the mean of each measure. They can also be paired, where they are measured from the same secondary osteon at once and the mean of individual ratios is calculated. We evaluate the impact of pooling and pairing on the resulting ratio values and their interpretation of bone remodelling.

**Materials and methods:**

Using both approaches, we calculated the area ratio of 551 Haversian canals and secondary osteons from measurements of existing midshaft femur histology slides. The sample represents *n* = 38 young and middle-aged females of different social backgrounds from a historical osteological collection. Univariate and bivariate statistics were used to compare and evaluate paired and pooled values and their differences.

**Results:**

The pooled method usually returned a lower canal-to-osteon value than paired data across the whole sample, ages, and social groups. These methods differed in the ratios they produced, independently of the number of measurements per sample.

**In conclusion:**

The pooling technique indicated the presence of thicker lamellar bone within secondary osteons, suggesting greater quantities of bone formed during BMU activity, than the paired technique results, which suggested less bone formed per BMU. While pooling offers efficiency in data collection and analysis, we show that paired data yield more biologically precise and analytically flexible results at an individual BMU level.

**Supplementary Information:**

The online version contains supplementary material available at https://doi.org/10.1007/s00774-026-01747-7.

## Introduction

Bone remodelling activity can be reconstructed from cortical bone histology by applying static histomorphometry which measures different geometric properties of secondary osteons and their microanatomical features (such as Haversian canals, lamellae, osteocyte lacunae) [1–3]. These data give measures of size and shape of secondary osteons at different stages of the bone remodelling cycle executed by Basic Multicellular Units (BMUs) [4]. It is a useful technique in analyses that deal with cortical bone health and metabolic diseases or bone biomechanical competence [5–7]. Changes in physiology and/or pathological processes of bone metabolism can impact bone remodelling by altering the quantity or quality of lamellar bone deposited within secondary osteons by the BMU [8, 9]. These changes in bone remodelling can have further effects on overall bone quality by reducing cortical bone area or volume [5, 10], and can be attributed to a number of factors including nutrition [11], activity [12], socioeconomic status [13], age [14], hormonal changes (specifically menopause), and osteoporosis [15]. For instance, in osteoporosis, menopause, and age-related bone-loss, cortical bone porosity can increase the Haversian canal size relative to secondary osteon area [10, 16]. This increased intra-cortical porosity can transition into trabecularisation of cortical bone (which can also be a result of other diseases) [17, 18]. Morphometric analyses of any changes in cortical bone histology can thus offer a wealth of information about the underlying bone metabolism and are applicable in a range of disciplines that deal with bone histology, including clinical, biomedical, and archaeological settings [2, 3, 13]. Ensuring that remodelling reconstructions are as accurate as possible is crucial in all these disciplines.

In terms of histomorphometric measurements, the perimeter of the cement line indicates the transition between osteoclast-mediated resorption and osteoblast deposition in bone remodelling [19]. The size of the Haversian canal area reflects the space for blood vessels and thus space that is not occupied by bone deposited by osteoblasts, but a measure of area that can be deducted from the secondary osteon area to provide a measure of lamellar thickness [20]. While these two types of area data can be understood independently, bone histologists often derive a ratio of Haversian canal area (H.Ar) to secondary osteon area (On.Ar) (H.Ar:On.Ar, or H.Ar/On.Ar × 100) to better characterise individual BMU activity [e.g. 12, 14, 21, 22]. For example, such derived parameters have been used to explore the allometric and spatial relationships between bone macroscopic form (represented by cortical bone thickness) and microscopic variation in secondary osteon morphology and morphometry in the femur and ribs of humans and other animals [12, 14, 21, 22]. Ratio calculations in cortical bone histology can also take the form of other parameters, such as Haversian canal perimeter to bone area (HC.Pm/B.Ar) or fraction of bone area (B.Ar/On.Ar). These parameters have been used where future comparative data from micro-computed tomography 3D scans might be acquired for cross-analysis with 2D histological ratio measures of osteon bone surface and volume [12, 14]. In this study, we focus on 2D area relationships in ground cortical bone histology sections only.

**Fig. 1:**
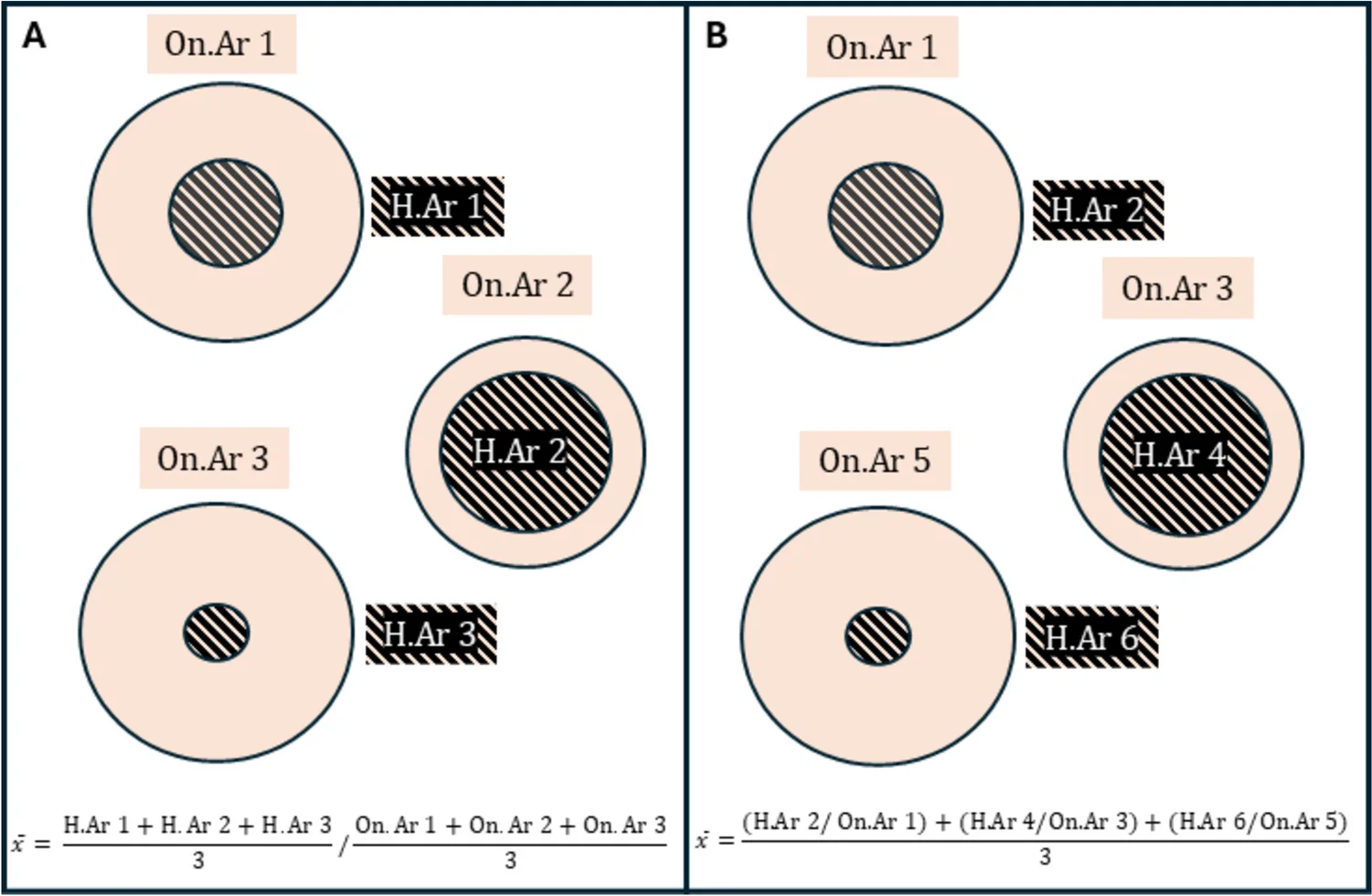
Schematic illustration of the two different techniques of obtaining Haversian canal (H.Ar) to secondary osteon area (On.Ar) ratio in a hypothetical cortical bone region of interest showing secondary osteons of variable geometric properties. **A** illustrates data pooling, whereas **B** illustrates data pairing. The numbers assigned to each secondary osteon and Haversian canal signify the sequence of collecting measurements. In **A**, all secondary osteon areas are collected separately from the Haversian canal areas. In **B**, the  secondary osteon area is collected first, followed by the area of its Haversian canal

During data collection, secondary osteon and Haversian canal size data can be obtained in a pooled or paired way (Fig. 1). When pooling, all secondary osteon and Haversian canal areas in a sample are collected independently, and the ratio is calculated from the arithmetic mean of each measure. When pairing, areas are collected and ratios calculated from the same osteon at the same time and then the arithmetic mean of all ratios in a sample is calculated. While both approaches appear to give a measure of the same BMU activity across a specified bone region, they use different mathematical operations and weighting principles. As has been shown in other disciplines and in fundamental statistics, data pooling shows the overall composition, weighted by size, whilst pairing shows the typical proportional relationship (ratio) where all structures are given equal weighting [23–25]. In other words, pooling works with the ratio of sums, whereas pairing works with the mean of ratios. Given these mathematical foundation differences, and unless all secondary osteons in a sample are the same size and perfectly proportional to their Haversian canals (which is highly unlikely in human bone), each technique will lead to different bone remodelling results. Here, we evaluate the impact of applying pairing vs pooling to secondary osteons within cortical bone on resulting ratio values. We examine this using a typical osteological sample size and discuss the ensuing interpretations of bone remodelling activity.

## Materials and methods

**Fig. 2:**
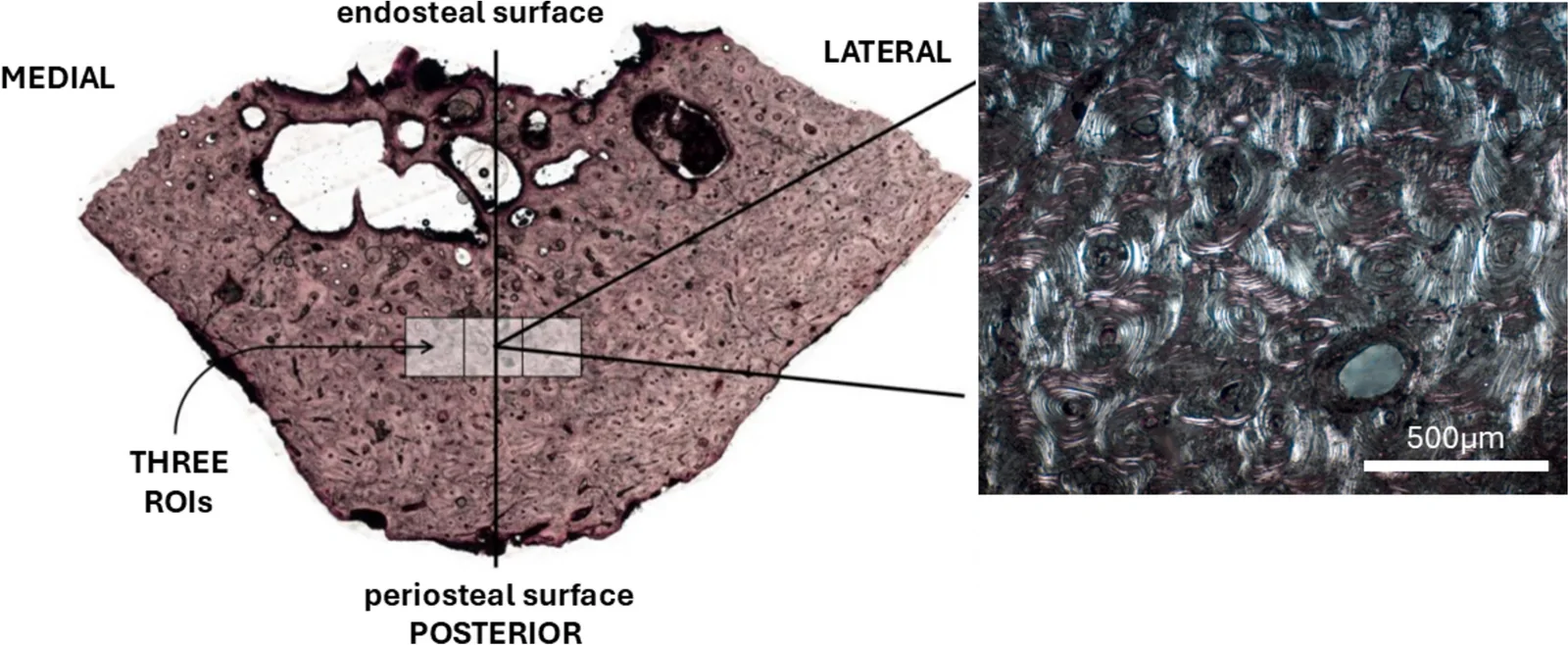
Stitched microscopic image of a representative bone sample from our study indicating the region of interest (ROI) location on the intra-cortical bone with an example of one ROI viewed at 50 × magnification

Posterior femur midshaft histology slides, which constituted part of an existing historical osteological collection (dated to the medieval period in England) curated at the University of Kent (Canterbury, UK), were examined. Permission for analyses of these slides was obtained from the collection curator, local ethics advisory board (November 2010), and Human Research Ethics Committee at the University of Queensland (2024/HE002340). This is a well-preserved collection of hundreds of human skeletal remains for which biological sex and age were estimated based on anthropological anatomical standards [26]. These histology slides were initially prepared for other research dealing with bone remodelling and anatomical and behavioural variation e.g. [13, 14, 27], which noted differences in bone remodelling between subgroups of distinct socioeconomic status (SES) (low SES characteristic of medieval poorer social class and high SES characteristic of privileged social classes). The slides have since been curated as a histological collection for future analyses as part of the larger osteological assemblage. A total of 38 slides, all of which represented females of young (20–34 years) and middle age (35–50 years), were randomly selected. They were subsequently blinded and given to another observer for imaging under an Olympus BX60 high powered microscope equipped with a MIchrome 5 Pro high-resolution camera. Each slide had three regions of interest (ROI) imaged intra-cortically at 50 × magnification from the centre of the sample and obtained by moving the microscope stage in consecutive distances of each ROI (Fig. 2).

Images were then analysed using ImageJ part of the FIJI package using the polygon tool tracing the cement line and canal border. A total of 551 secondary osteons were measured using the paired and pooling technique, with averages calculated by the arithmetic mean:

Pooling technique: $R pool=\frac{\overline{\mathcal{C}} }{\overline{O} },$ where $\overline{\mathcal{C} }= \frac{1}{n} \sum_{i=1}^{n}{C}_{i}$ and $\overline{O }= \frac{1}{n} \sum_{i=1}^{n}{O}_{i,}$

Pairing technique: $$R paired=\frac{1}{n}\sum_{i=1}^{n}\frac{{C}_{i}}{{O}_{i}},$$

where *n* = count of values, *O*_*i*_ = is the *i*th secondary osteon area, and *C*_*i*_ = is the *i*th Haversian canal area.

Not all samples had the same number of secondary osteons measured due to inconsistencies in cortical bone preservation and thus visibility of clear cement lines. Twenty per cent of the sample was evaluated for intra-observer error rate through a technical error of measurement (TEM) [28], by calculating the difference between original and repeated measurements and the coefficient of reliability. Using PAST software (vol. 5.3 October 2025; [29]), data means (reported along with confidence intervals [CI]) were calculated per sample (from across all three ROIs) and compared using a Wilcoxon Signed Rank test (reporting the smaller of the two rank sums as W) as the pooled data group was not normally distributed. In addition, due to the uneven number of secondary osteons measured per sample, we ran a Spearman’s Rho correlation test to check whether sampling size or density of measurement has an effect on the relationship between the ratios produced by each method. To visualise data distribution, On.Ar and H.Ar were plotted against the difference between ratio methods (Δ = Paired ratio – pooled ratio) in R [30], using packages readxl [31], ggplot2 [32], tidyverse [33], and ggbeeswarm [34]. Within each individual, correlations of raw measurements between On.Ar and H.Ar, and between H.Ar/On.Ar and On.Ar, were also tested with Spearman’s Rho (with a Bonferroni correction) in PAST 5.3 (Supplementary Table 1).

## Results

The intra-observer TEM was low with a high coefficient of reliability (> 0.9), suggesting overall consistency between repeated measurements. The individual area and ratio data for each sample are shown in Table 1, where it is evident that there are differences in the mean values when comparing pooled and paired ratios. The mean difference was 0.539, with the paired method generally giving a higher ratio value (paired mean = 8.989, CI =  ± 0.957; pooled mean = 8.450, CI =  ± 1.029), but it was also as high as 4.525 and as low as −1.820 in some cases. This difference was statistically significant (*W* = 168, *p* = 0.003) in the entire sample.

**Table 1:** Averaged data for each histology slide (*n* = 38) with the age (Y: young 20–34 years, M: middle aged 35–49 years) number of secondary osteons measured (N.On), Haversian canal (H.Ar), and secondary osteon area (On.Ar) in µm^2^ with standard deviation (SD), the ratios (H.Ar/On.Ar × 100), and the difference between the two ratio methods (Ratio Δ = Paired—Pooled)

Age	N.On	H.Ar	H.Ar SD	On.Ar	On.Ar SD	Pooled Ratio	Paired Ratio	Ratio Δ (Paired – Pooled)
Y	19	2257.279	963.044	32550.597	15724.611	6.935	8.725	1.790
M	15	7829.464	6716.292	45260.663	25542.316	17.299	16.307	−0.991
M	5	3425.524	549.009	26657.740	5144.524	12.850	13.146	0.296
M	8	4082.083	2872.604	49275.680	17902.837	8.284	9.972	1.688
Y	26	1507.715	1414.002	23513.205	18021.258	6.412	7.186	0.774
M	22	1788.983	1014.858	34576.147	16272.629	5.174	6.018	0.844
Y	7	2093.785	877.807	48198.916	21036.226	4.344	5.235	0.891
M	15	5111.097	3994.941	40967.305	14360.504	12.476	11.627	−0.849
Y	8	2366.626	1313.936	42833.476	18283.938	5.525	6.050	0.525
Y	20	3006.486	2228.563	38742.785	14620.158	7.760	8.778	1.018
Y	6	2030.615	1479.613	37102.687	17963.849	5.473	5.096	−0.377
M	6	4267.946	2866.403	49692.125	33529.163	8.589	8.998	0.409
M	16	5121.225	3761.775	47361.419	26429.995	10.813	11.174	0.360
M	14	1478.413	971.095	26171.295	16016.028	5.649	6.393	0.744
Y	12	1960.750	1078.752	34182.594	15577.821	5.736	6.693	0.957
Y	4	2845.203	3056.272	49947.699	10384.085	5.696	5.353	−0.343
Y	9	3172.116	1562.140	37524.631	18792.253	8.453	9.287	0.834
Y	12	1843.633	1511.344	35149.353	22112.213	5.245	5.726	0.481
Y	15	2202.893	1572.478	25490.787	14530.093	8.642	8.430	−0.212
M	27	2319.061	1890.799	22171.186	12589.991	10.460	10.045	−0.415
Y	28	2635.921	1350.384	28103.962	14556.153	9.379	10.310	0.931
M	7	1808.331	960.339	34324.240	21755.654	5.268	7.337	2.069
M	14	6360.523	4970.275	47369.403	27760.661	13.427	13.565	0.137
Y	19	3134.415	2755.772	40533.505	31660.261	7.733	7.683	−0.049
M	24	5878.056	5781.921	34598.054	21546.656	16.990	17.046	0.057
Y	21	3325.068	2283.253	45182.213	21309.063	7.359	7.709	0.349
Y	8	2736.452	3293.106	44530.576	33582.290	6.145	4.963	−1.182
Y	8	3898.753	2058.892	48105.867	26687.472	8.105	8.578	0.474
Y	8	1629.883	894.751	20272.785	20506.504	8.040	12.565	4.525
M	19	2640.382	2122.407	37400.608	17387.941	7.060	7.726	0.666
Y	11	3443.879	2505.058	39048.553	15705.279	8.819	8.672	−0.147
Y	26	2038.183	1088.144	24541.387	11867.522	8.305	9.599	1.294
M	12	2878.375	2986.223	39297.993	18069.525	7.324	8.062	0.737
M	16	3484.309	3666.148	42429.937	22365.051	8.212	8.330	0.118
M	17	5548.561	8602.995	39722.149	31536.622	13.968	12.148	−1.820
M	10	3905.761	2694.939	46113.188	23572.107	8.470	11.058	2.588
Y	15	3373.165	1929.048	52651.324	27035.849	6.407	7.767	1.360
M	22	2237.201	1917.178	27087.981	15293.875	8.259	8.209	−0.050

**Fig. 3:**
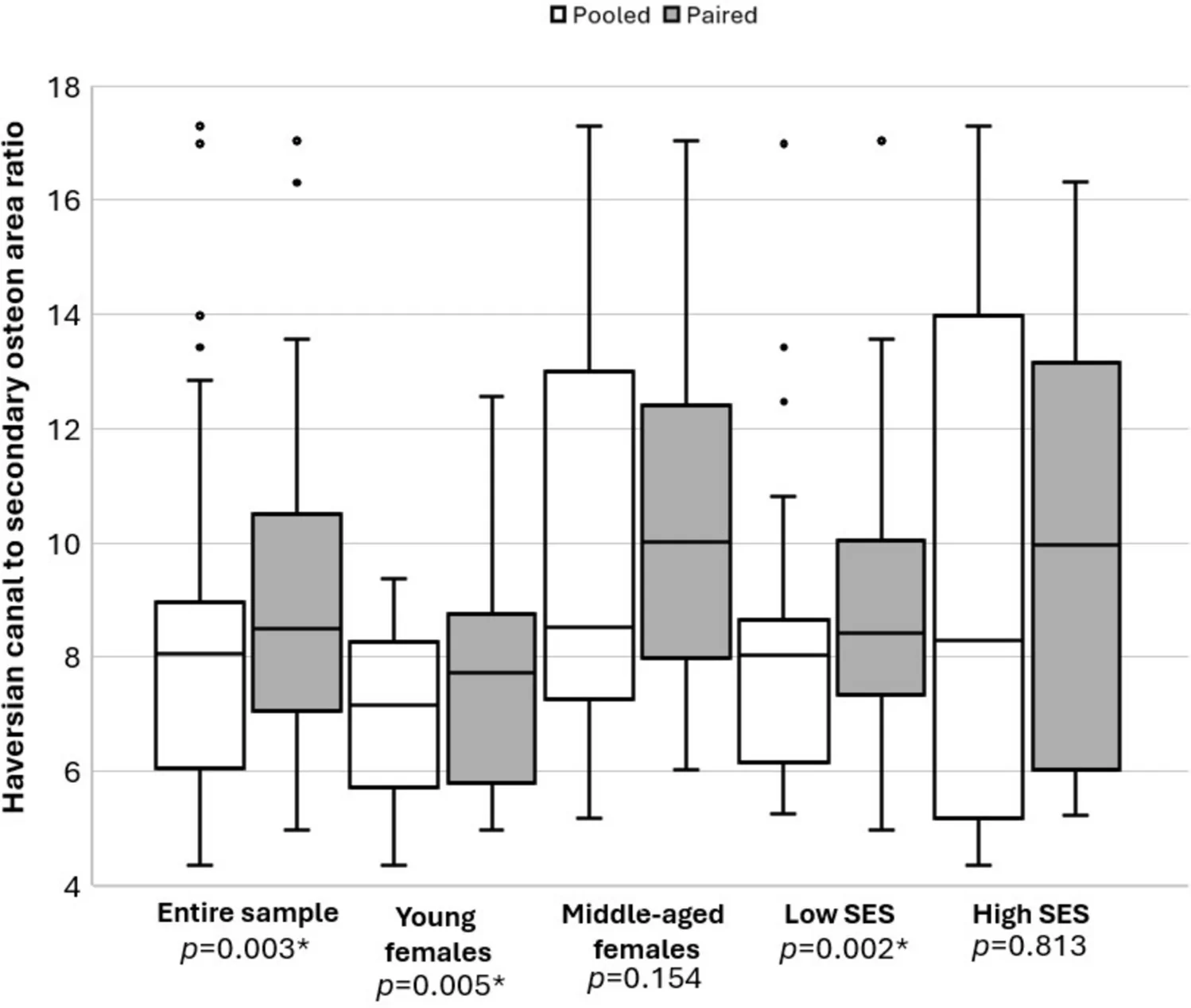
Box plots illustrating differences between Haversian canal-to-secondary osteon area ratio values obtained using the pooling vs pairing technique across age and socioeconomic status (SES) subgroups. Black dots indicate outliers. **p < 0.05

The trend for these results remained when splitting the dataset by ages, and social background (Fig. 3), in young females (*n* = 20; mean paired = 7.720, CI =  ± 0.929; mean pooled = 7.026, CI =  ± 0.665; *W* = 32; *p* = 0.005), middle-aged females (*n* = 18; mean paired = 10.398, CI =  ± 1.576; mean pooled = 10.032, CI =  ± 1.863; *W* = 52; *p* = 0.154), in the low SES (*n* = 31; mean paired = 8.760, CI =  ± 0.956; mean pooled = 8.153, CI =  ± 0.947; *W* = 94; *p* = 0.002), and high SES (*n* = 7; mean paired = 10.002, CI =  ± 3.790; mean pooled = 9.762, CI =  ± 4.591; *W* = 12; *p* = 0.813) subgroups.

**Fig. 4:**
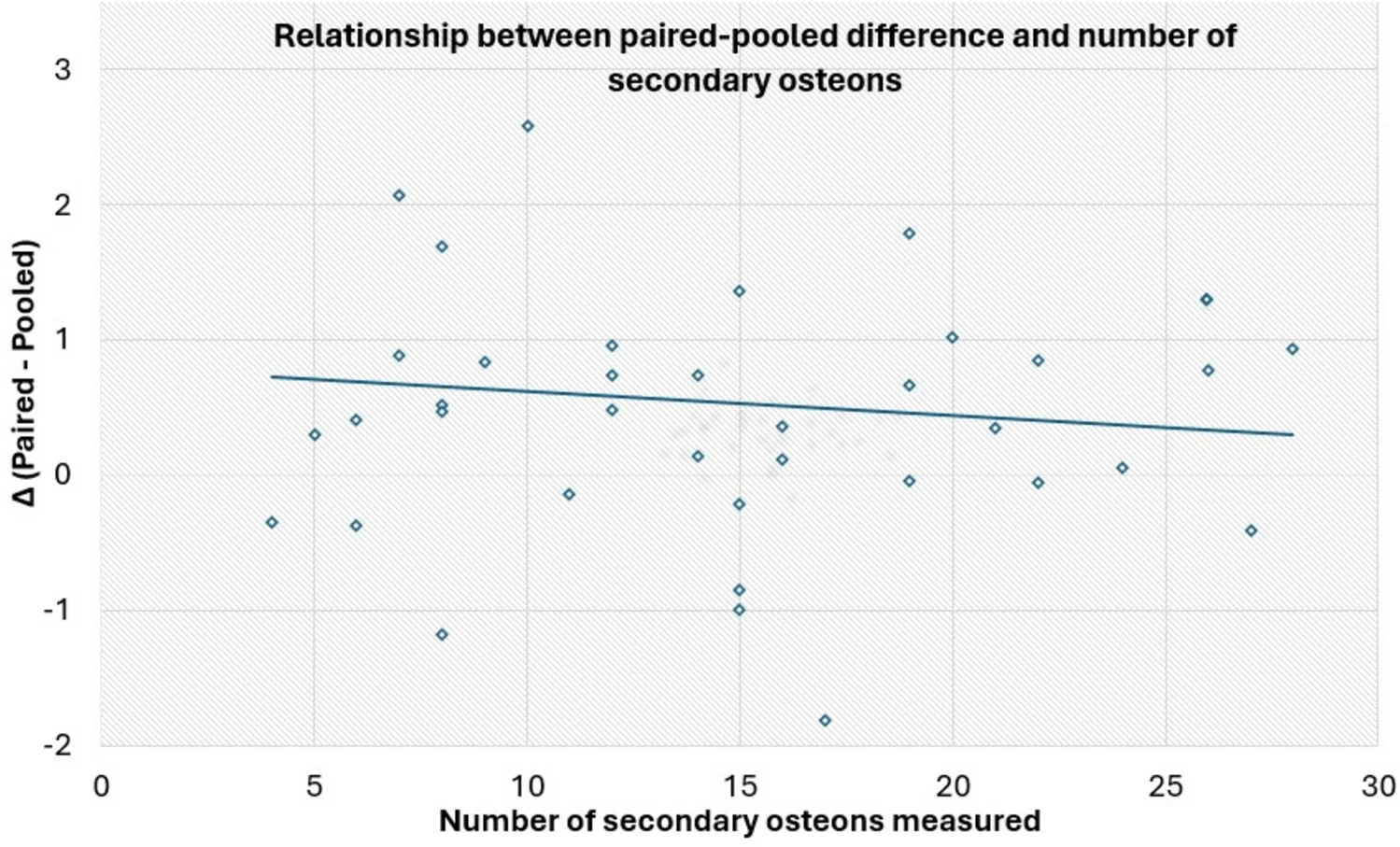
Scatterplot correlating the number of secondary osteons measured per sample with the paired-pooled ratio difference value (Rho = −0.026, *p* = 0.879)

**Fig. 5:**
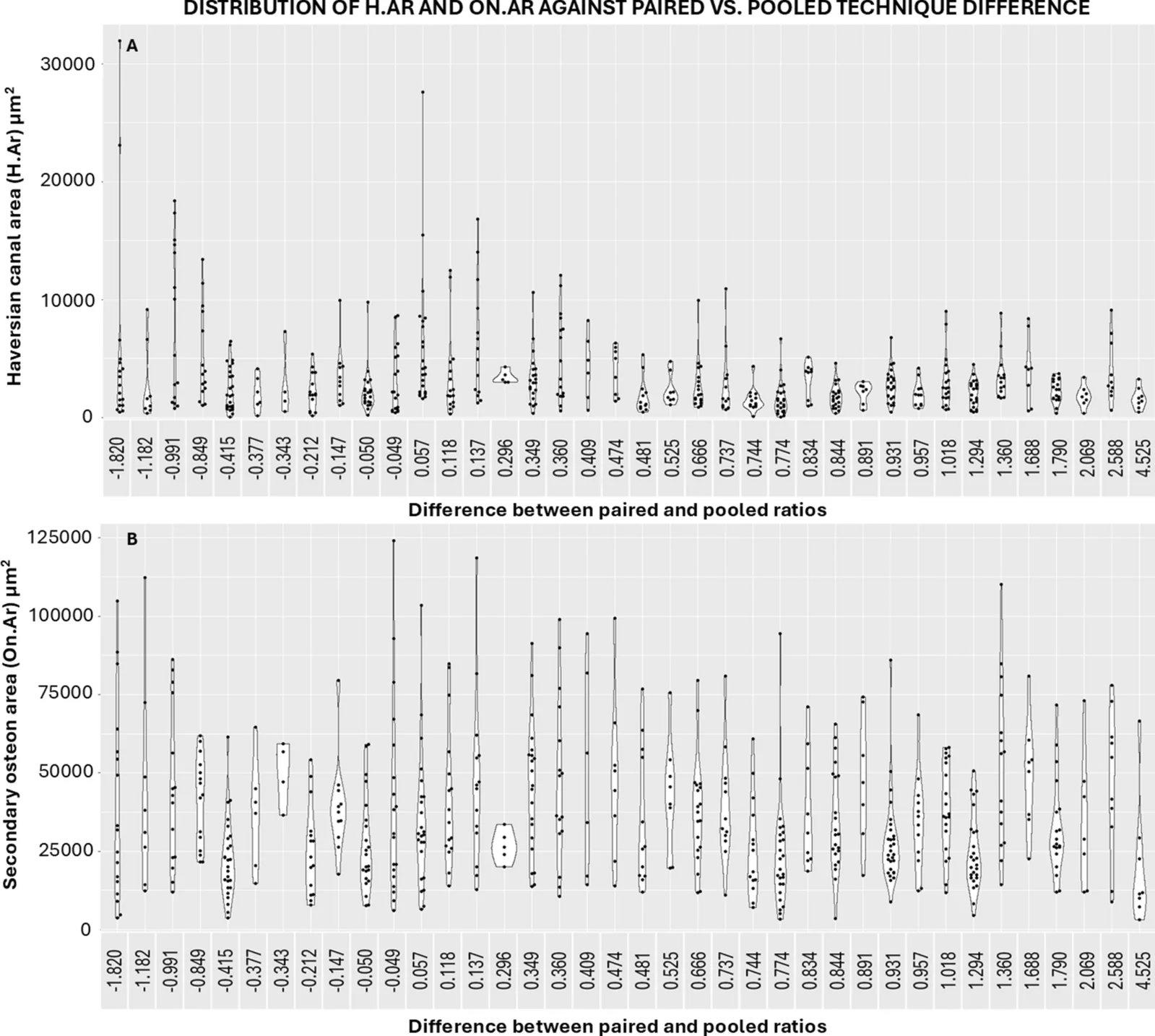
Distribution of Haversian canal (H.Ar) (**A**) and secondary osteon area (On.Ar) (**B**) against the difference in paired and pooled ratios showing that increased H.Ar area and distribution of measurements was associated with a negative difference in ratio, and thus higher ratios when calculated by pooling. It should be noted that the x-axis is not to scale, with the ratio differences given for each individual in ascending order

The Spearman’s Rho correlation between the number of secondary osteons measured per sample and the paired-pooled difference value was not statistically significant (*p* = 0.879) and had a very low correlation coefficient (Rho = −0.026) (Fig. 4). The distribution of H.Ar and On.Ar against the difference in paired and pooled rations is illustrated in Fig. 5, showing some variability in H.Ar associated with positive and negative ratio differences. Negative and low positive ratios had wider H.Ar distributions than those with larger positive differences (Fig. 5a), while On.Ar distributions showed no difference associated with the difference in pooled and paired ratio difference (Fig. 5b). The Spearman’s Rho correlations per each individual (Supplementary Table 1) showed that those with negative Δ had higher Spearman’s Rho values on average.

## Discussion with conclusions

The ratio values of Haversian canal-to-secondary osteon area will always be different if calculated through independent versus paired averaging during data collection and analysis. In our sample, using pooled averages of secondary osteon and Haversian canal areas to calculate the ratio of canal space to lamellar bone typically resulted in a lower value than that calculated by averaging the ratios of individual remodelling events from paired Haversian canals and secondary osteon areas. This difference in the calculated ratios has implications for existing and future interpretations about bone remodelling from such ratio results, highlighting that researchers must be aware of caveats around interpretations of such ratios, and ought to explicitly state which averaging technique had been applied in their analysis.

Because the ratio value is treated as an index of whether the proportion of Haversian canal space to secondary osteon space is low or high and thus signifying the proportional amount of lamellar bone present in each secondary osteon, the higher the index, the lower amounts of lamellar bone. Since this index varies depending on the averaging approach, reconstructions of the speed of bone remodelling or its dynamics linked to known variables or context (health, disease, biomechanics) will usually be higher when the data are paired. Moreover, in the pooling technique, outlier (usually larger) secondary osteons are weighted more heavily than under calculations using the paired technique.

In one of the samples examined here, the difference between the two distinct averaging approaches was as high as 4.525, with the pooled ratio returning a value of 8.040 compared to a paired ratio value of 12.565. While such disparate results might not always be the case, if they do occur, they may lead to inaccurate or inconsistent conclusions about bone remodelling, particularly when compared across a sample or in relation to a baseline in a given population.

**Fig. 6:**
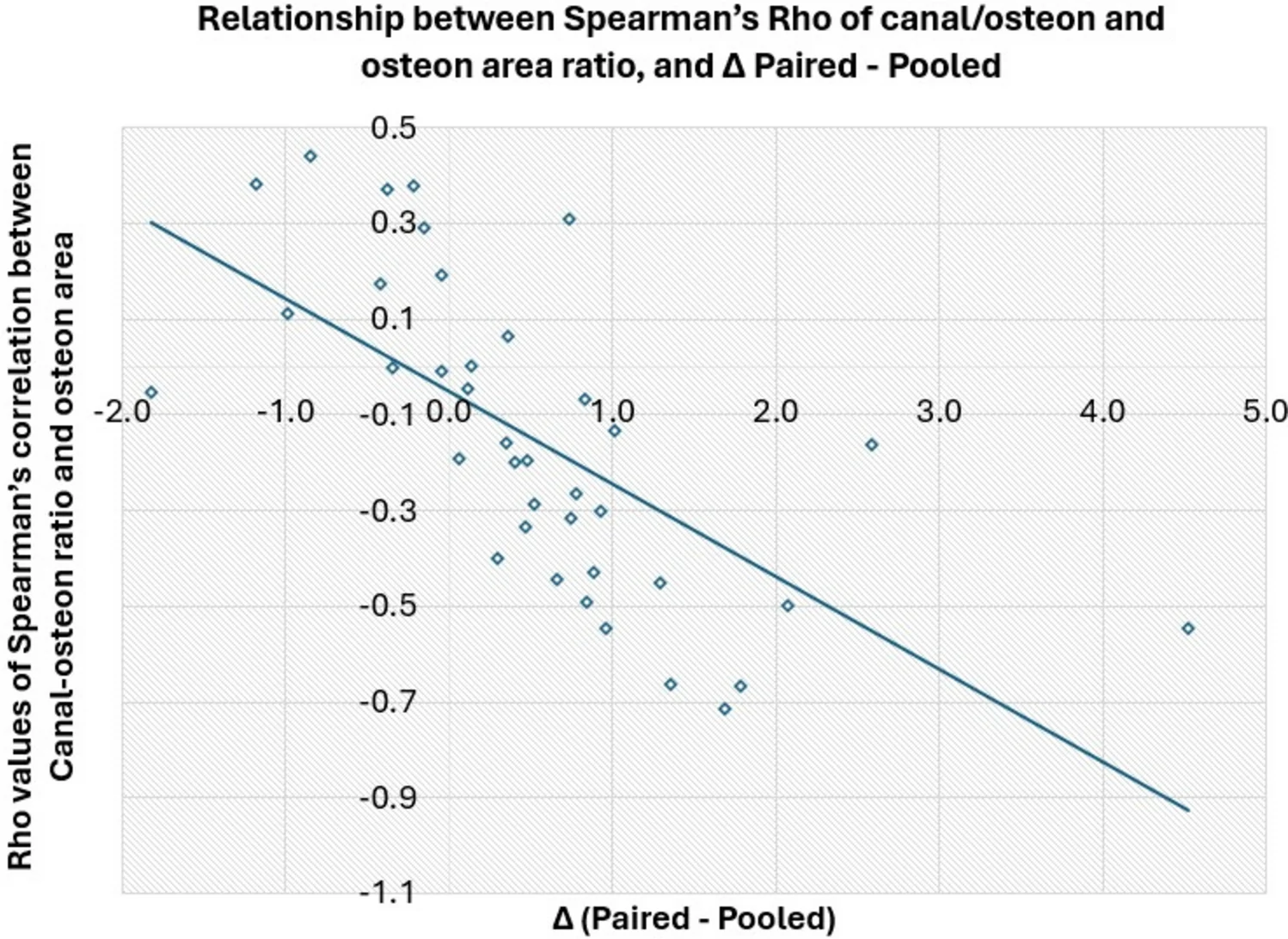
Illustration of the relationship between Spearman’s Rho values of canal-to-secondary osteon area ratio and secondary osteon area correlation against the difference values (Δ) of the paired vs pooled method

Although the trend within our sample showed a reduction in the ratio when calculated using the pooling method, this was not universal, with 11 individuals showing a higher ratio when calculated through pooled averages. The difference between methods (Δ = paired – pooled) for these 11 was not as extreme (−1.820) as seen in those where the paired method had higher ratios (the greatest difference of 4.525). Nor were they of a particular age group, split evenly between young and middle-aged adults. These results are possibly explained by Chebyshev’s sum inequality, which states that for non-decreasing ${x}_{i}$ and ${y}_{i}$, then $\frac{1}{n}\sum {x}_{i}{y}_{i}\ge \left(\frac{1}{n}\sum {x}_{i}\right)\left(\frac{1}{n}\sum {y}_{i}\right)$. Substituting ${x}_{i}=\frac{{C}_{i}}{{O}_{i}}$ and ${y}_{i}={O}_{i}$ yields $\frac{1}{n}\sum {C}_{i}\ge \left(\frac{1}{n}\sum \frac{{C}_{i}}{{O}_{i}}\right)\left(\frac{1}{n}\sum {O}_{i}\right)$, and because $\left(\frac{1}{n}\sum {O}_{i}\right)>0$ will always be true for our data, this can be divided from both sides, producing Rpool≥Rpaired. Biological data such as ours are unlikely to ever be monotonic, but examining the Spearman’s Rho correlation of the relationship between $\frac{{C}_{i}}{{O}_{i}}$ and ${O}_{i}$ indicates that the 11 individuals with negative Δ had higher Spearman’s Rho values on average (Fig. 6), and thus tend towards being monotonic. These individuals also had higher significant Spearman’s Rho correlations between H.Ar and On.Ar (Supplementary Table 1). Together, this indicates that in individuals where the variables of interest are isometric or positively allometric, then the pooled method will always give higher values than the paired method. Conversely, where the variables of interest are not strongly correlated, isometric, or positively allometric, such as most individuals examined here, the paired method can produce higher values. Interestingly, the individuals with negative Δ were predominantly of low SES, although with a small sample size it is not possible to determine if this is a statistical artefact or a real biological phenomenon.

Beyond the natural variability in secondary osteon and Haversian canal size, the range in the calculated ratios we noted is likely a reflection of the demographic spread of our sample. The age of the females included in our study likely had a significant impact on bone quality, with some females in the middle age category possibly experiencing menopause prior to their death, which would impact their Haversian canal area [7, 10, 14, 16, 35], increasing the ratios observed. Indeed, the females with the highest ratios were from the older middle-aged category, and the smallest ratios came from those in the young adult subgroup. To add further variability to these results, previous studies, including of this sample from Canterbury, have noted the impacts of SES on bone remodelling [13, 36], noting high SES to be associated with larger H.Ar, possibly due to a less active lifestyle than that of the lower social classes that engaged in strenuous physical labour [13]. As we examined a sample of histology slides from an anthropological collection with sexes and ages estimated from macroscopic skeletal features, we are limited in further exploring specific biological factors underlying the variation seen in the ratios. Further research with a larger, more demographically diverse cohort of documented age, sex, and other biological factors may provide more detailed insight into how the pooled and paired methods perform.

As large, outlier secondary osteons bias the mean of the pooled ratio, as demonstrated by the association between high average H.Ar and pooled ratio noted previously, the paired method can be considered more precise, as indicated by the smaller confidence intervals for the paired results across the entire sample in our study. In recording individual secondary osteons in the paired method, we can obtain individual BMU activity information which provides greater flexibility for further analysis than pooling. Ratio data for individual secondary osteons/remodelling events can be later paired with additional analyses such as osteocyte density or mineralisation of lamellar bone within specific secondary osteons. We acknowledge that in the use of published or preexisting datasets, only aggregate data may be available. This should not dissuade researchers from using the pooled method to calculate secondary osteon ratios, only that users of this method should be cognisant of the potential limitations and skews in the results.

We recognise that efficiency in data collection is an important factor of any research project, particularly in analyses of bone histology [37, 38] and pooling values may be easier than pairing when working with larger sample sizes. The paired method requires additional time spent on data collection to ensure that canals and secondary osteons are correctly paired and in extracting individual measurements from image analysis software. Conversely, the pooling method does not require correct association or collection order of Haversian canals or secondary osteons. Although the pooled method may be only slightly quicker in data collection per one individual, a small increase in time for the paired method is accumulative and can result in a significant increase in data collection time for large datasets. Furthermore, the subsequent data analysis and management is more efficient for the pooled method, with only mean aggregates calculated and collected, and fewer calculations required for the resulting ratio than that of the paired method.

While not affecting all individuals within our dataset, the general trend whereby pooling results in lower ratios should be noted with care when used for interpretations of bone remodelling across different populations and comparisons between studies. When used as an indicator of issues with bone remodelling, such as in investigations of osteoporosis or associations with SES, the use of the pooling method may understate increased porosity due to disrupted remodelling, particularly where the correlations between H.Ar and On.Ar are positive and significant. Thus, it will be important to explore data both at an individual and entire sample level, as supported by our identification of individual-level variation in ratios. When undertaking comparisons between studies that have used different methods, data from individuals where pooling had been applied may indicate lower intra-cortical porosity, but it could be an artefact of the pooling method. We thus emphasise the importance of stating which method is applied, so that comparisons may be made accurately and interpretations made with care. In some of the prior literature examining the Haversian canal-to-secondary osteon ratio [12, 14, 21, 22], it has not been explicitly stated how the ratio had been treated, but for the two publications we authored [21, 22], we confirm we pooled the data for secondary osteon and Haversian canal area. Future research should consider pairing these size measures or at least be explicit in how the means were calculated. Moreover, future studies could examine which technique most closely correlates with individual BMU activity through experimental treatment of modern observations, as well as potentially examining other ways that the means could be calculated. For example, the harmonic mean might be a more appropriate or accurate average to calculate for ratios such as the ratio of Haversian canal-to-secondary osteon area.

Unless all or most secondary osteons measured are of the same dimensions, there will always remain statistical skew in these ratio values. As such, it would be prudent to also provide measures of data spread (i.e. variance) in measured datasets. Further testing and validation of these statistical properties in different populations would be worthwhile, since our sample represents Caucasian individuals, all estimated to be biologically female, from a single historical collection. However, this should not pose a great problem to the significance of the technical aspects of the work. We also specifically considered females because they are commonly investigated in bone biology around reproductive system effects on bone remodelling. Testing for any differences between averaging techniques on males may provide further perspectives on this technical issue.

## Supplementary Information

Below is the link to the electronic supplementary material.

Supplementary file1 (DOCX 27 KB)

## Data Availability

Averages per individual are provided in the article, while all raw data points can be accessed open access from figshare: https://doi.org/10.6084/m9.figshare.31810003.
