# Supplementary material for: Evaluating Haversian canal-to-osteon ratios for reconstructing bone remodelling activity: balancing precision and efficiency in data collection
Source: J Bone Miner Metab. 2026 Aug 10;44(5):646–55. doi: 10.1007/s00774-026-01747-7 (PMC13723443; doi:10.1007/s00774-026-01747-7)
Supplement: Supplementary file 1 [file 774_2026_1747_MOESM1_ESM.docx]

**SUPPLEMENTARY MATERIAL FOR**

**Evaluating Haversian canal-to-osteon ratios for reconstructing bone remodelling activity: balancing precision and efficiency in data collection**

Karen M. Cooke^1*^, Isabella Pearce^2^, R. Claire Aland^2^, Julien Louys^3^, Patrick Mahoney^4^, Justyna J. Miszkiewicz^1,5*^

^1^ School of Social Science, University of Queensland, St Lucia, Queensland, Australia

^2^ School of Biomedical Sciences, University of Queensland, St Lucia, Queensland, Australia

^3^ Australian Research Centre for Human Evolution, Griffith University, Nathan, Queensland, Australia

^4^ School of Natural Sciences, University of Kent, Canterbury, UK

^5^ Earth, Life, Time Group, Naturalis Biodiversity Center, Leiden, The Netherlands

Corresponding authors: [karen.cooke@uq.edu.au](mailto:karen.cooke@uq.edu.au); [j.miszkiewicz@uq.edu.au](mailto:j.miszkiewicz@uq.edu.au)

**SUPPLEMENTARY TABLE**

**Supplementary Table 1**. Results from Spearman Rho correlations within each individual for relationships between between raw Haversian canal and secondary osteon area, and ratio of Haversian canal to secondary osteon area and secondary osteon area. N.On: number of secondary osteons per individual, H.Ar: Haversian canal area, On.Ar: secondary osteon area. *statistically significant at Bonferroni corrected *p*=0.00132.

| **N.On** | **H.Ar** | **On.Ar** | **Pooled Ratio** | **Paired Ratio** | **Δ (Paired - Pooled)** | **Spearman's Rho**  **H.Ar vs On.Ar** | **Rho *p* value**  **H.Ar vs On.Ar** | **Spearman's Rho**  **H.Ar/On.Ar vs On.Ar** | **Rho *p* value**  **H.Ar/On.Ar vs On.Ar** |
| --- | --- | --- | --- | --- | --- | --- | --- | --- | --- |
| 17 | 5548.561 | 39722.149 | 13.968 | 12.148 | -1.820 | 0.843 | <0.0001* | -0.054 | 0.837 |
| 8 | 2736.452 | 44530.576 | 6.145 | 4.963 | -1.182 | 0.929 | 0.002 | 0.381 | 0.360 |
| 15 | 7829.464 | 45260.663 | 17.299 | 16.307 | -0.991 | 0.600 | 0.018 | 0.111 | 0.694 |
| 15 | 5111.097 | 40967.305 | 12.476 | 11.627 | -0.849 | 0.746 | 0.001 | 0.439 | 0.101 |
| 27 | 2319.061 | 22171.186 | 10.460 | 10.045 | -0.415 | 0.773 | <0.0001* | 0.175 | 0.384 |
| 6 | 2030.615 | 37102.687 | 5.473 | 5.096 | -0.377 | 0.771 | 0.081 | 0.371 | 0.497 |
| 4 | 2845.203 | 49947.699 | 5.696 | 5.353 | -0.343 | 0.600 | 0.417 | 0.000 | 1.000 |
| 15 | 2202.893 | 25490.787 | 8.642 | 8.430 | -0.212 | 0.775 | 0.0007* | 0.379 | 0.164 |
| 11 | 3443.879 | 39048.553 | 8.819 | 8.672 | -0.147 | 0.773 | 0.005 | 0.291 | 0.385 |
| 22 | 2237.201 | 27087.981 | 8.259 | 8.209 | -0.050 | 0.844 | <0.0001* | -0.010 | 0.966 |
| 19 | 3134.415 | 40533.505 | 7.733 | 7.683 | -0.049 | 0.849 | <0.0001* | 0.191 | 0.433 |
| 24 | 5878.056 | 34598.054 | 16.990 | 17.046 | 0.057 | 0.690 | <0.0002* | -0.190 | 0.373 |
| 16 | 3484.309 | 42429.937 | 8.212 | 8.330 | 0.118 | 0.453 | 0.078 | -0.047 | 0.863 |
| 14 | 6360.523 | 47369.403 | 13.427 | 13.565 | 0.137 | 0.692 | 0.006 | 0.002 | 0.994 |
| 5 | 3425.524 | 26657.740 | 12.850 | 13.146 | 0.296 | 0.300 | 0.683 | -0.400 | 0.483 |
| 21 | 3325.068 | 45182.213 | 7.359 | 7.709 | 0.349 | 0.634 | 0.002 | -0.160 | 0.489 |
| 16 | 5121.225 | 47361.419 | 10.813 | 11.174 | 0.360 | 0.568 | 0.022 | 0.065 | 0.812 |
| 6 | 4267.946 | 49692.125 | 8.589 | 8.998 | 0.409 | 0.714 | 0.119 | -0.200 | 0.714 |
| 8 | 3898.753 | 48105.867 | 8.105 | 8.578 | 0.474 | 0.762 | 0.037 | -0.333 | 0.428 |
| 12 | 1843.633 | 35149.353 | 5.245 | 5.726 | 0.481 | 0.636 | 0.026 | -0.196 | 0.542 |
| 8 | 2366.626 | 42833.476 | 5.525 | 6.050 | 0.525 | 0.595 | 0.124 | -0.286 | 0.501 |
| 19 | 2640.382 | 37400.608 | 7.060 | 7.726 | 0.666 | 0.302 | 0.209 | -0.444 | 0.057 |
| 12 | 2878.375 | 39297.993 | 7.324 | 8.062 | 0.737 | 0.510 | 0.090 | 0.308 | 0.331 |
| 14 | 1478.413 | 26171.295 | 5.649 | 6.393 | 0.744 | 0.600 | 0.023 | -0.314 | 0.274 |
| 26 | 1507.715 | 23513.205 | 6.412 | 7.186 | 0.774 | 0.549 | 0.004 | -0.264 | 0.193 |
| 9 | 3172.116 | 37524.631 | 8.453 | 9.287 | 0.834 | 0.617 | 0.086 | -0.067 | 0.880 |
| 22 | 1788.983 | 34576.147 | 5.174 | 6.018 | 0.844 | 0.497 | 0.018 | -0.491 | 0.020 |
| 7 | 2093.785 | 48198.916 | 4.344 | 5.235 | 0.891 | 0.393 | 0.375 | -0.429 | 0.354 |
| 28 | 2635.921 | 28103.962 | 9.379 | 10.310 | 0.931 | 0.391 | 0.040 | -0.299 | 0.122 |
| 12 | 1960.750 | 34182.594 | 5.736 | 6.693 | 0.957 | 0.147 | 0.649 | -0.545 | 0.067 |
| 20 | 3006.486 | 38742.785 | 7.760 | 8.778 | 1.018 | 0.293 | 0.210 | -0.132 | 0.578 |
| 26 | 2038.183 | 24541.387 | 8.305 | 9.599 | 1.294 | 0.437 | 0.025 | -0.452 | 0.021 |
| 15 | 3373.165 | 52651.324 | 6.407 | 7.767 | 1.360 | 0.214 | 0.443 | -0.664 | 0.007 |
| 8 | 4082.083 | 49275.680 | 8.284 | 9.972 | 1.688 | -0.405 | 0.327 | -0.714 | 0.058 |
| 19 | 2257.279 | 32550.597 | 6.935 | 8.725 | 1.790 | -0.009 | 0.972 | -0.665 | 0.002 |
| 7 | 1808.331 | 34324.240 | 5.268 | 7.337 | 2.069 | 0.286 | 0.527 | -0.500 | 0.267 |
| 10 | 3905.761 | 46113.188 | 8.470 | 11.058 | 2.588 | 0.539 | 0.108 | -0.164 | 0.651 |
| 8 | 1629.883 | 20272.785 | 8.040 | 12.565 | 4.525 | 0.452 | 0.255 | -0.548 | 0.171 |
